# Impurities in amyloid studies: The power of automated model building within a cautionary tale for structural biologists

**DOI:** 10.1002/pro.70353

**Published:** 2025-10-22

**Authors:** David Rhyner, Lukas Frey, Jiangtao Zhou, Witek Kwiatkowski, Raffaele Mezzenga, Roland Riek, Jason Greenwald

**Affiliations:** ^1^ Institute of Molecular Physical Science ETH Zürich Zürich Switzerland; ^2^ Department of Health Sciences and Technology ETH Zurich Zurich Switzerland

**Keywords:** amyloid materials, amyloids, CryoEM, human lysozyme, ModelAngelo, *Oryza sativa*

## Abstract

The purity of protein samples of biological origin is often difficult to ascertain, leading the naïve or optimistic scientist to underestimate contaminants in their research. Even after extensive purification, protein samples can contain nucleic acids, truncated degradation products, or other protein contaminants. While in many cases, and when present at low concentrations, such contaminants are unlikely to alter experimental results significantly, they must be considered when studying protein aggregation. Such reactions can be sensitive to small environmental changes in their early stages due to a nucleation‐dependent mechanism, where minor differences can be amplified during the subsequent exponential growth phase. During a recent study of the amyloid formation of human lysozyme, we encountered a significant amyloid‐forming protein contaminant derived from the expression host *Oryza sativa japonica*. Further investigation of this widely used commercial source of human lysozyme revealed at least a dozen protein contaminants. These discoveries led to intriguing observations, including an underdeveloped branch of plant amyloid research and a possible link between the amyloid fold and allergens. Here, we present our findings within a cautionary tale for structural biologists: a surprising variety of contaminants in a commercial protein sample and the accidental yet definitive identification of one of them by cryo‐electron microscopy helical reconstruction. The resulting 2.54 Å model of the 17 kDa alpha‐amylase/trypsin inhibitor Type 2 marks the first known amyloid structure of a plant protein.

## 1 INTRODUCTION

Recent advances in cryo‐electron microscopy (CryoEM) single‐particle reconstruction have revolutionized structural biology, enabling near‐atomic resolution imaging of complex biomolecules and supramolecular assemblies of the type previously not amenable to straightforward structural analyses (Kühlbrandt, 2014). Among these are amyloids, highly ordered, β‐sheet‐rich protein aggregates associated with neurodegenerative diseases such as Alzheimer's and Parkinson's (Iadanza et al., 2018; Sawaya et al., 2021). However, amyloid research extends beyond disease‐associated systems; amyloids with diverse functions have been discovered in nature (Otzen & Riek, 2019), are actively explored for their potential as materials (Li et al., 2014) and catalysts (Marshall & Korendovych, 2021), and are hypothesized to have played a role in the origin of life (Carny & Gazit, 2005; Greenwald et al., 2018; Maury, 2009; Orgel, 2008). The in vitro study of amyloid structures with CryoEM typically follows a well‐established workflow (Fromm & Sachse, 2016; He & Scheres, 2017). Each step presents technical challenges, yet one critical issue is often overlooked: the role of impurities in influencing experimental outcomes and reproducibility. Due to their instability and origin from complex mixtures, biological samples are rarely as pure as typical chemical reagents used in the lab, and the difficulty in assaying their purity often leaves researchers in the position of having to assume that their samples are “pure enough.” The flip side is that even trace impurities can significantly alter experimental results, a particularly relevant problem for amyloid studies. It has been suggested that virtually any protein can adopt an amyloid fold under the right conditions, heightening the risk of interference from other protein impurities (Chiti & Dobson, 2006; Goldschmidt et al., 2010; Rousseau et al., 2006). Small environmental changes can also significantly impact experimental outcomes due to the auto‐catalytic nature of amyloid aggregation, rendering amyloid research especially vulnerable to impurities, proteinaceous or otherwise. As a case in point, our investigation into a widely used, commercially sourced human lysozyme revealed an unexpected contaminant: the 17 kDa alpha‐amylase/trypsin inhibitor Type 2 (UniProt ID: AI172), a protein native to the expression host, *Oryza sativa japonica*, and similar in size to the 14,692 Da lysozyme (Frey et al., 2024). Importantly, this contaminant went unnoticed until we had solved the structure of what we at first thought were lysozyme amyloid fibrils. Eventually, we could “sequence” these amyloids using high‐resolution CryoEM maps as input to the automated model‐building program ModelAngelo (Jamali et al., 2024), allowing us to identify the contaminant. Further investigations into the sample composition revealed a dozen other proteinaceous impurities from the expression host and led us to a few potentially interesting observations. Among the impurities, we found that only the amyloidogenic AI172 belongs to a group of known allergens in *O. sativa japonica* (Adachi et al., 1993; Alvarez et al., 1995). This immunogenic function may be related to its propensity to form amyloid fibrils (Pérez‐Tavarez et al., 2021). Interestingly, we could find no other amyloid structures of plant proteins deposited in the electron microscopy data bank (EMDB), even though the amyloid fold is expected to be equally prevalent in plant proteins. This apparent neglect of plant amyloids is likely due to the historical association of amyloids with human diseases. However, functional plant amyloids involved in seed protein storage have recently been documented (Antonets et al., 2020; Santos & Ventura, 2021) and there is increasing interest in understanding the roles/uses of amyloids in the food industry (Li et al., 2020; Nikbakht Nasrabadi et al., 2021; Pang et al., 2020). Furthermore, with the growing interest in amyloids in materials science, plant proteins will be a sustainable and inexpensive source of amyloid fibrils (Knowles & Mezzenga, 2016; Li et al., 2023; Wei et al., 2017; Zhou et al., 2022). Our findings underscore two key points: first, the importance of vigilance when analyzing protein structures at moderate resolutions where misinterpretations are more likely, and second, the utility of automated machine learning‐based tools like ModelAngelo to mitigate human biases and detect errors that might otherwise be overlooked.

## 2 RESULTS AND DISCUSSION

### 2.1 Identification of an unknown protein in CryoEM data of human lysozyme fibrils

**FIGURE 1:**
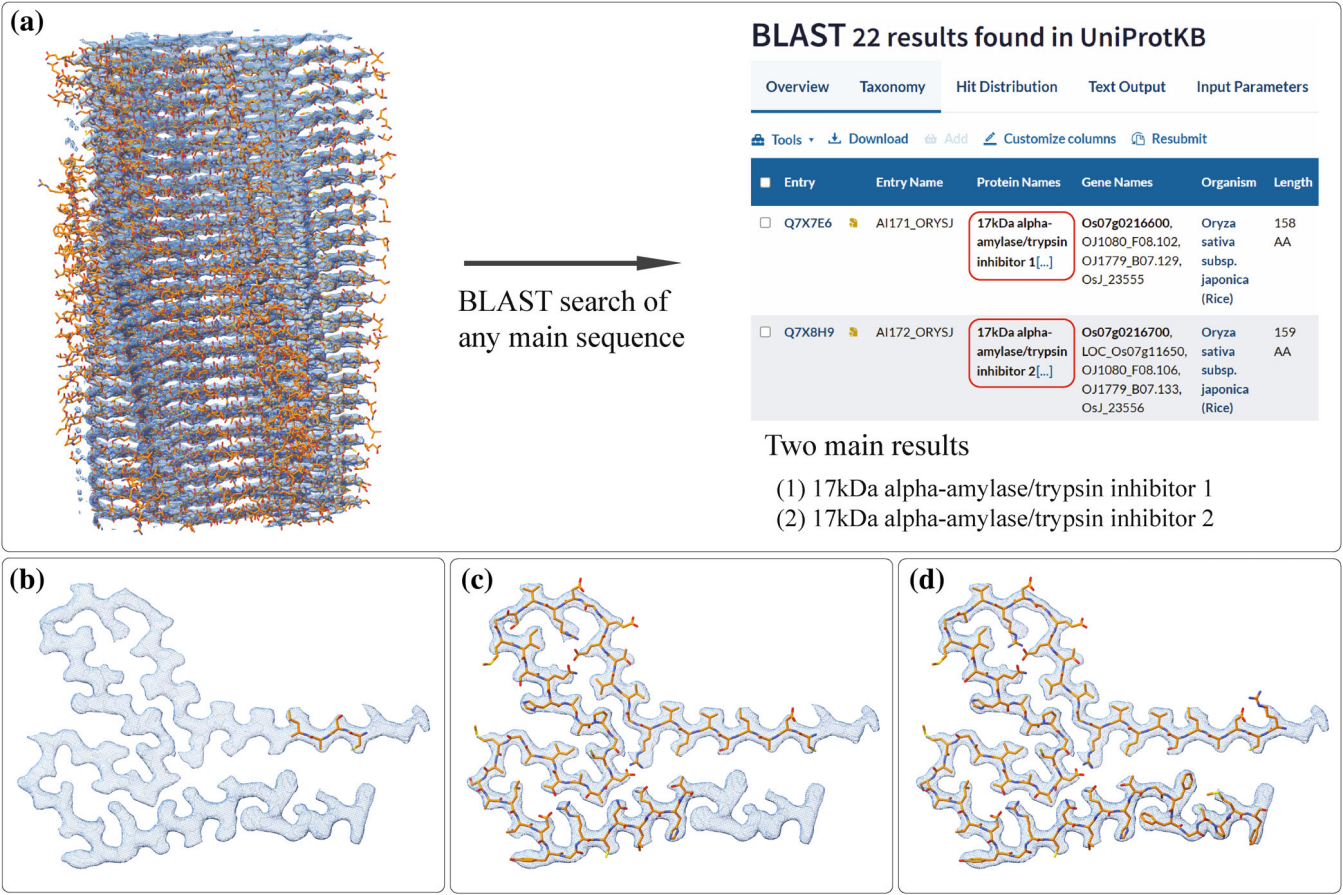
Identification of an unknown protein using ModelAngelo. (a) A BLAST search with any of the output sequences generated by running ModelAngelo without a sequence input (model_angelo build_no_seq) resulted in two clear hits: 17 kDa alpha‐amylase/trypsin inhibitor Type 1 (AI171) and 17 kDa alpha‐amylase/trypsin inhibitor Type 2 (AI172) from *Oryza sativa japonica* (rice). Output models from running ModelAngelo with (a) human Lysozyme as the input sequence, (c) AI171 as the input sequence, and (d) AI172 as the input sequence.

In a recently published article on reversible versus irreversible amyloids (Frey et al., 2024), we examined human lysozyme amyloid fibrils using CryoEM. In one set of micrographs from a single fibril sample, we quickly detected two distinct protein amyloids in the 2D classes. As the lysozyme sample had been obtained from a commercial source, we mistakenly assumed that we were dealing with two distinct structural polymorphs of the same human lysozyme. However, despite having reached a high resolution for one of the CryoEM reconstructions, we could not manually fit the sequence of human lysozyme into the density map. This led us to suspect that a different protein had been isolated in the CryoEM analyses, so we turned to automated map‐fitting to try to determine the sequence of the protein. We chose ModelAngelo (Jamali et al., 2024), a program based on a trained neural network developed in the Scheres lab and the latest in a series of fully automated model‐building programs (Chang et al., 2022; Chojnowski et al., 2022; He et al., 2022; Hoh et al., 2020; Liebschner et al., 2019; Nakamura et al., 2023; Pfab et al., 2021; Terashi & Kihara, 2018; Terwilliger et al., 2021; Zhang et al., 2022). Somewhat satisfying to the human researchers trying to put a square peg into a round hole, ModelAngelo also failed to yield a model when human lysozyme was used as the input sequence (Figure 1b). However, running ModelAngelo without an input sequence yielded nearly complete models in which each layer of the amyloid was fit with unique but similar sequences. Taking any of these sequences as input for a basic local alignment search tool (BLAST) search against the Swissprot database resulted in two clear hits: 17 kDa alpha‐amylase/trypsin inhibitor Type 1 (AI171) and 17 kDa alpha‐amylase/trypsin inhibitor Type 2 (AI172) from *O. sativa japonica* (Figure 1a). While both sequences were recognized at around 70%–80% sequence identity, depending on which sequence layer from the ModelAngelo output was used, AI172 consistently scored higher than AI171. Also, AI172 yielded more complete models than AI171 when running ModelAngelo with each of the two sequences as inputs (Figure 1c,d). This is striking because for the 67 residues visible in the amyloid fold, AI171 and AI172 have 92.5% sequence identity, differing only at the following five positions: A112S, an insertion of alanine between residues T146 and G147, F148S, G150A, and P152L. The product specifications of the commercial human lysozyme (Sigma Aldrich L1667) that we used indicate that it was indeed produced in *O. sativa*, thereby providing a logical explanation for our results.

### 2.2 The amyloid fold of the plant protein AI172 from *Oryza sativa japonica*

**FIGURE 2:**
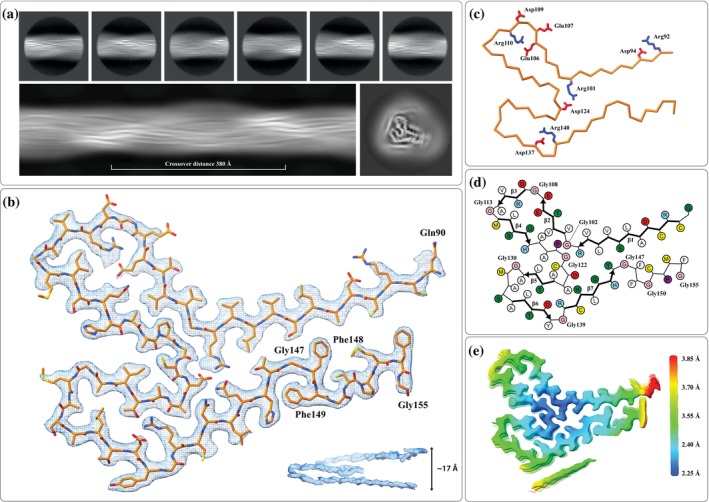
(a) 2D Class averages (*top*) were used to calculate an initial de novo 3D model (*bottom*). (b) The cryo‐electron microscopy (CryoEM) density in the region of a single chain of the atomic model of AI172, with inlay showing the side view of one layer of the CryoEM density, illustrating how a single chain ascends through several 4.75 Å repeats. (c) The Cα trace highlighting negatively charged (Glu and Asp) in red and positively charged (Arg) side chains in blue, including three buried salt bridges (i.e., Arg101/Asp124, Arg110/Glu106, and Arg140/Asp137). (d) Schematics highlighting amino acids and β‐sheets segments (β1–β7). The schematics were produced using atom2svg.py (Nakane, 2020). Positively charged residues in blue, negatively charged residues in red, polar residues in green, nonpolar residues in white, sulfur‐containing residues in yellow, and glycines in pink. (e) CryoEM map color‐coded by local resolution including the unmodeled density displayed below the amyloid core.

AI172 is a 159‐residue (135‐residue without signal peptide) protein from the rice plant *O. sativa japonica*. For the CryoEM analysis, the segments belonging to AI172 were identified using 2D classification. The resulting 2D class averages were used to calculate a de novo initial 3D model with an approximate crossover distance of 315 Å (Figure 2a), which was then used as input to perform automated 3D refinement, resulting in a high‐resolution map (Figure S1). The automated model building described above yielded the atomic model depicted in Figure 2b which shows that the C‐terminal region of AI172 (residues Gln90‐Gly155) is involved in the formation of the amyloid fibril core. In addition, there is a strand‐like density that was not modeled due to its low quality (visible in Figure 2e) but is likely from the N‐terminal region of the sequence. Three inter‐strand salt bridges (i.e., Arg101/Asp124, Arg110/Glu106, and Arg140/Asp137) are buried inside the fold (Figure 2c). Turns between strands are often at the positions of flexible glycine residues (i.e., Gly102, Gly108, Gly113, Gly122, Gly130, Gly139, Gly147, and Gly150). The parallel beta‐sheet segments (β1‐β7) are formed by residues Cys91‐Arg101 (β1), Val103‐Glu106 (β2), Asp109‐Ala112 (β3), Met114‐His118 (β4), Ala126‐Ala129 (β5), Ala134‐Tyr138 (β6), and Arg140‐Tyr146 (β7) (Figure 2d). Coloring the CryoEM map by local resolution shows the highest resolution around the fibril's rotational axis and a gradually lower resolution toward its surface (Figure 2e). The segment Gly147‐Phe148‐Phe149 (labeled in Figure 2b) forms an unusual double aromatic motif. Another distinctive feature of the amyloid fold of AI172 is that a single chain ascends through several layers of the cross‐β motif, such that the intersheet packing within the fold often occurs between different neighboring strands (up to four chains away). The side view of a single chain shows this unusual feature and how a single chain spans 17 Å along the length of the fibril instead of the typical 4.75 Å (shown in Figure 2b).

### 2.3 Analysis by mass spectrometry verifies the presence of AI172 and reveals several other protein impurities originating from *Oryza sativa japonica*

**FIGURE 3:**
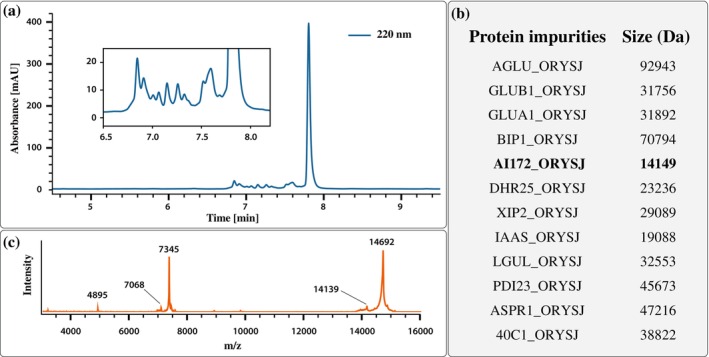
(a) The reverse‐phase HPLC chromatogram of a denatured human lysozyme sample reveals many proteinaceous impurities eluting around the lysozyme peak. (b) Protease digestion followed by LC–MS/MS analysis of the same sample revealed several proteinaceous impurities from the expression host, including the amyloid‐forming AI172. Protein sizes were calculated for reduced cysteines. (c) MALDI‐MS spectra. The peaks around 4895 and 7345 are triply and doubly charged ions of the full‐length human lysozyme (Mw ≈14,692 Da). The peak around 7068 is the doubly charged ion of the full‐length impurity AI172 (Mw ≈14,139 Da).

Identifying a significant amyloid‐forming impurity in our human lysozyme sample motivated us to pursue further analysis. We first performed reverse‐phase high performance liquid chromatography (HPLC) analysis of a fibrillized human lysozyme sample (incubated 20 mg/mL, 100 mM and 1,4‐dithiothreitol (DTT), 10 mM NaCl, 3 h at 85°C, and then denatured in 6M guanidine). Estimating the purity by peak integration of the 220 nm absorbance signal is consistent with the reported value, “higher than 90% by sodium dodecyl sulfate–polyAcrylamide gel electrophoresis (SDS‐PAGE)” reported in the product specification (Figure 3a). The same sample was then digested using chymotrypsin and analyzed by timsTOF LC–MS/MS. The resulting masses were evaluated with the Mascot software against the SwissProt database. Using a high standard for protein identification that requires at least five significant unique fragment sequences, 16 unique proteins were identified. Apart from human lysozyme, chymotrypsin (added for the analysis), and two human contaminants (albumin and keratin), the remaining 12 proteins originated from the expression host *O. sativa* (Figure 3b). Further analysis of the commercial human lysozyme sample by matrix‐assisted laser desorption/Ionization mass spectrometry (MALDI‐MS) (before fibrillation and DTT treatment) revealed an impurity peak at a molecular weight of 14,139 Da (Figure 3c). With its five intact disulfide bridges, this corresponds well to AI172 (fully reduced mass of 14,149 Da). Although AI172 is potentially the most abundant impurity protein, this data suggests that it accounts for significantly less than 10% of the sample composition. However, in the CryoEM analyses, approximately 21% of the fibril segments that displayed a significant twist belonged to the AI172 fold. That what by HPLC and MS appears to be a minor contaminant could become significantly overrepresented in the CryoEM sample can have several causes, some related to the nature of EM sample preparations and some specific to amyloid aggregation and analysis. Better initial solubility, preferential fibrilization at the given conditions, and better freezing behavior might have all contributed to the overrepresentation of amyloids from an impurity. In addition to the relative overrepresentation of AI172 in the sample, AI172 also gave rise to data of higher quality, which led unsuspecting researchers like us to select it in the initial screening of the 2D class averages. Thus, what is likely a lower structural heterogeneity in the AI172 amyloid fibrils not only led to their passive selection in the analysis but also to a higher final resolution for AI172 of 2.54 Å, compared to the 2.80 Å resolution of the human lysozyme amyloid fibrils from the same set of micrographs.

### 2.4 Speculation on the potential role of amyloids in food allergenicity

AI172 belongs to the alpha‐amylase/trypsin inhibitor family and has been identified as a rice allergen (Adachi et al., 1993; Kurokawa et al., 2013; Satoh et al., 2011). This raises the question of whether there is a potential connection between AI172's propensity to form amyloid fibrils and its allergenicity. It has been hypothesized that the stability of specific food allergens may result from their ability to form stable amyloid fibrils. The fibrillar form of the allergen may enhance immune responses by increasing the binding affinity of IgE antibodies (Pérez‐Tavarez et al., 2021). For example, Gad m 1 (β‐parvalbumin) causes allergic reactions only after aggregation into an amyloid fibril (Martínez et al., 2015). For some food allergens, such as Bos d 5 (β‐lactoglobulin), Bos d 10 (κ‐casein), Bos d 12 (αs2‐casein), Gal d 2 (ovalbumin), and Gal d 4 (lysozyme), amyloid formation is well documented (Cao & Mezzenga, 2019; Goers et al., 2002; Pan & Zhong, 2015; Sánchez et al., 2016; Thorn et al., 2008). On the other hand, a recent safety study of amyloid fibrils of Bos d 5 (β‐lactoglobulin) and Gal d 4 (lysozyme) showed lower levels of immunogenicity than their native monomeric forms, suggesting the opposite to be the case for these two proteins specifically, or perhaps even generally for pre‐hydrolyzed amyloid proteins (Xu et al., 2023), since hydrolyzed proteins are well‐known to possess lower allergenicity than full sequence parent proteins. We hope that our results will further encourage researchers to investigate this potential connection and its nuances further.

## 3 CONCLUSION

The current advanced state of biological techniques, especially in proteomics and high‐resolution mass spectrometry, leaves little excuse for not knowing your protein sample in intimate detail. The traditional phrase “single band by SDS‐PAGE” applied to purified proteins is non‐quantitative and outdated. The field of amyloids and protein aggregation suffers from poor reproducibility across experiments and research groups, including variations in kinetic profiles and differences in polymorphism and polymorph distribution. Much of this irreproducibility might be avoided if researchers work with purer and, thereby, more similar protein samples. We found that AI172 easily formed stable amyloid fibrils. It was also the only protein among all the contaminants known as an allergen. This further supports a previously suggested link between amyloid formation and allergic reactions. As AI172 can, in principle, be produced cheaply in large quantities, it could potentially be a valuable system in the field of amyloid‐based materials.

## 4 METHODS

### 4.1 Materials

Human lysozyme (Sigma Aldrich L1667), Chymotrypsin (Sigma Aldrich C4129), DTT (D0632) were purchased from Sigma Aldrich. The AI172 and the rigid type of human lysozyme fibrils were formed by incubating soluble human lysozyme (20 mg/mL lysozyme at pH 7 and filtered with a 0.45 μm filter, 100 mM DTT, 10 mM NaCl) for 3 h at 85°C with magnetic stirring at 300 rpm. A more detailed procedure can be found in the original lysozyme publication (Frey et al., 2024).

### 4.2 Chymotrypsin digestion of human lysozyme rigid fibrils

A representative sample (40 μL) of rigid human lysozyme fibrils was added to guanidine (8 M, 140 μL) in a 1.5 mL Eppendorf tube and incubated for 30 min at room temperature. A total protein concentration of 340 μM was determined from the absorbance at 278 nm and an extinction coefficient of human lysozyme of 38,940/cm M. For protease digestion, the denatured protein solution diluted to 50 μM in 50 mM Tris pH 7.4 with 1.2 M residual guanidine was incubated with chymotrypsin (1 μM) with shaking at 37°C for 16 h. The digested sample was then analyzed by liquid chromatography‐tandem mass spectrometry (LC–MS/MS).

### 4.3 HPLC analysis

Reverse‐phase analysis of the denatured (but non‐digested sample) was performed on a 2.6 μm 4.6 × 150 mm Kinetex polar C18 column (Phenomenex) connected to an Agilent 1200 HPLC system equipped with an autosampler and diode array detector and resolved using a linear acetonitrile gradient with 0.1% trifluoroacetic acid (35%–55%) at a 1.35 mL/min flow rate. The main peak area was used to quantify the lysozyme via its extinction coefficient at 280 and 220 nm. The minor peak areas were used to estimate the lysozyme purity by assuming all contaminants have the same extinction coefficient as lysozyme at 220 nm.

### 4.4 LC–MS/MS analysis

LC–MS/MS was performed on a Bruker trapped ion mobility spectrometry‐time‐of‐flight (timsTOF) Pro 1 instrument equipped with an Ion Mobility‐Q‐Time‐Of‐Flight (IM‐Qq‐TOF) mass analyzer using Electro‐Spray Ionization (ESI). A trap column (Thermo Trap Cartridge 5 mm) was used to desalt the sample, followed by a separation column (PepSep 25 Series, 150 μm, 1.5 μm). Two microliters of digested human lysozyme sample was loaded, followed by a linear acetonitrile gradient with 0.1% formic acid (2%–35%) at a 0.30 μL/min flow rate.

### 4.5 Method for matrix‐assisted laser desorption ionization mass spectrometry

Human lysozyme solution (30 mg/mL) was desalted using C18 Zip Tips (Millipore, USA) and spotted onto the MALDI target (Microtitration plate 384 target polished steel TF, Bruker Daltonics, Bremen, Germany) along with a solution of ‐cyano‐4‐hydroxycinnamic acid (‐CHCA, 2 μL, 10 mg/mL) in acetonitrile/water/trifluoroacetic acid (50:50:0.1). MALDI measurements were performed on an ultra fleXtreme matrix‐assisted laser desorption/ionization time‐of‐flight/time‐of‐flight mass spectrometer equipped with a smart beam laser (Bruker Daltonics). The measurement parameters were programmed in flexControl (Version 3.4) in the positive linear mode with acquisition ranging from 600 to 30,000 Da. Final spectra consisted of 1000–3000 shots per analysis.

### 4.6 MASCOT search and protein identification

The Mascot MS/MS ion search was performed on the SwissProt database using the Mascot Daemon extension and a peak list file (.mgf) as input. The taxonomy option was set to “All entries,” quantitation and crosslinking were set to “None,” and no modifications were selected. Chymotrypsin was chosen for the enzyme option, while up to three missed cleavages were allowed. We searched for monoisotopic mass values with peptide and fragment tolerances of 20 ppm and selected electrospray ionization quadrupole time‐of‐flight as the instrument option. The search results were further filtered by requiring at least five significant, unique sequences to identify a protein.

### 4.7 Electron microscopy grid preparation and data collection

Cu R2/2300 mesh grids (Quantifoils) were glow discharged at 25 mA for 30 s. Freshly glow‐discharged grids were used in a Vitrobot Mark IV (Thermo Fisher Scientific) with its chamber set at 100% humidity and at a temperature of 15°C. Fibrils (4 μL aliquots) were applied to the grid and blotted for 5 s using a blot force of 1 after a 5‐s wait time and subsequently plunge‐frozen into a liquid ethane/propane mix. The grids were clipped and immediately used or stored in liquid nitrogen. Data acquisition was performed on a Titan Krios (Thermo Fisher Scientific) operating at 300 kV equipped with a Gatan Imaging Filter (GIF) with a 20 eV energy slit using Gatan's K3 direct electron detector in counting mode. Movies of 40 frames were collected using EPU software (Thermo Fisher Scientific) at a magnification of 130 kx and a dose rate of approximately 8 e/pix/s, and a total dose of ca. 63 e/Å^2^ in counted super‐resolution mode at a pixel size of 0.325 Å and binned to 0.65 Å.

### 4.8 Image processing

Image processing and helical reconstruction were carried out with RELION 4.0, following the procedure for amyloid structures described by Scheres (Scheres, 2020). RELION's motion correction was used to correct for drift and dose‐weighting, and contrast transfer function (CTF) estimation was done using Ctffind4.1 (Rohou & Grigorieff, 2015). Individual filaments were selected using auto‐picking, and segments were extracted in a 333 Å box with an inter‐box distance of 33 Å and binned 4× to a pixel size of 2.6 Å for initial 2D classification (see Table S1 for details). From an initial set of 1,836,771 segments, we manually selected 667,448 segments corresponding to 2D classes resembling twisted fibrils. Of these, we separated 140,376 segments (21%) from classes distinctly different from those of human lysozyme. We then used *relion_helix_inidmod2d* to generate an initial model that could be used for the subsequent 3D refinement steps. For the final 3D refinements and CTF refinement, segments were extracted at a pixel size of 0.65 Å.

### 4.9 Model building and refinement

Models were built into the RELION post‐processed maps with ModelAngelo (Frey et al., 2024). The output was manually adjusted in COOT (Emsley & Cowtan, 2004) and further adjusted by real‐space refinement as a nine‐layer fibril in ISOLDE (Croll, 2018) with symmetry and secondary structure restraints. The outer four layers, which often diverge slightly in structure due to their placement at the model's edges, were removed for deposition of the central five layers in the Protein Data Bank. Figures were prepared with CCP4MG (McNicholas et al., 2011) and University of California San Francisco (UCSF) Chimera (Pettersen et al., 2004).

## AUTHOR CONTRIBUTIONS

**David Rhyner:** Conceptualization; investigation; writing – original draft; methodology; validation; visualization; writing – review and editing. **Lukas Frey:** Methodology; investigation; supervision; validation. **Jiangtao Zhou:** Investigation; writing – review and editing; validation; methodology. **Witek Kwiatkowski:** Methodology; investigation; validation. **Raffaele Mezzenga:** Funding acquisition; writing – review and editing; validation; investigation. **Roland Riek:** Funding acquisition; investigation; conceptualization; writing – review and editing; validation. **Jason Greenwald:** Writing – review and editing; investigation; conceptualization; methodology; validation; supervision; funding acquisition.

## Supporting information


**Data S1.** Supporting Information.

## Data Availability

The reconstructed CryoEM map was deposited in the Electron Microscopy Data Bank with the accession code EMD‐53234. The coordinates of the atomic model were deposited in the Protein Data Bank (PDB) under the accession code PDB 9QLU.
