# Supplementary material for: Impurities in amyloid studies: The power of automated model building within a cautionary tale for structural biologists
Source: Protein Sci. 2025 Oct 22;34(11):e70353. doi: 10.1002/pro.70353 (PMC12542297; doi:10.1002/pro.70353)
Supplement: Supplementary file 1 — Data S1. Supporting Information. [file PRO-34-e70353-s001.docx]

Supporting information for

Impurities in Amyloid Studies: The Power of Automated Model Building within a Cautionary Tale for Structural Biologists

David Rhyner^1^, Lukas Frey^1^, Jiangtao Zhou^2^, Witek Kwiatkowski^1^, Raffaele Mezzenga^2^, Roland Riek^1^,*, Jason Greenwald^1^,*

1. Institute of Molecular Physical Sciences, ETH Zürich, Vladimir-Prelog-Weg 2, CH-8093 Zürich, Switzerland
2. Department of Health Sciences and Technology, ETH Zurich, Zurich, Switzerland.

*Corresponding authors: [roland.riek@phys.chem.ethz.ch](mailto:roland.riek@phys.chem.ethz.ch),

[jason.greenwald@phys.chem.ethz.ch](mailto:jason.greenwald@phys.chem.ethz.ch),


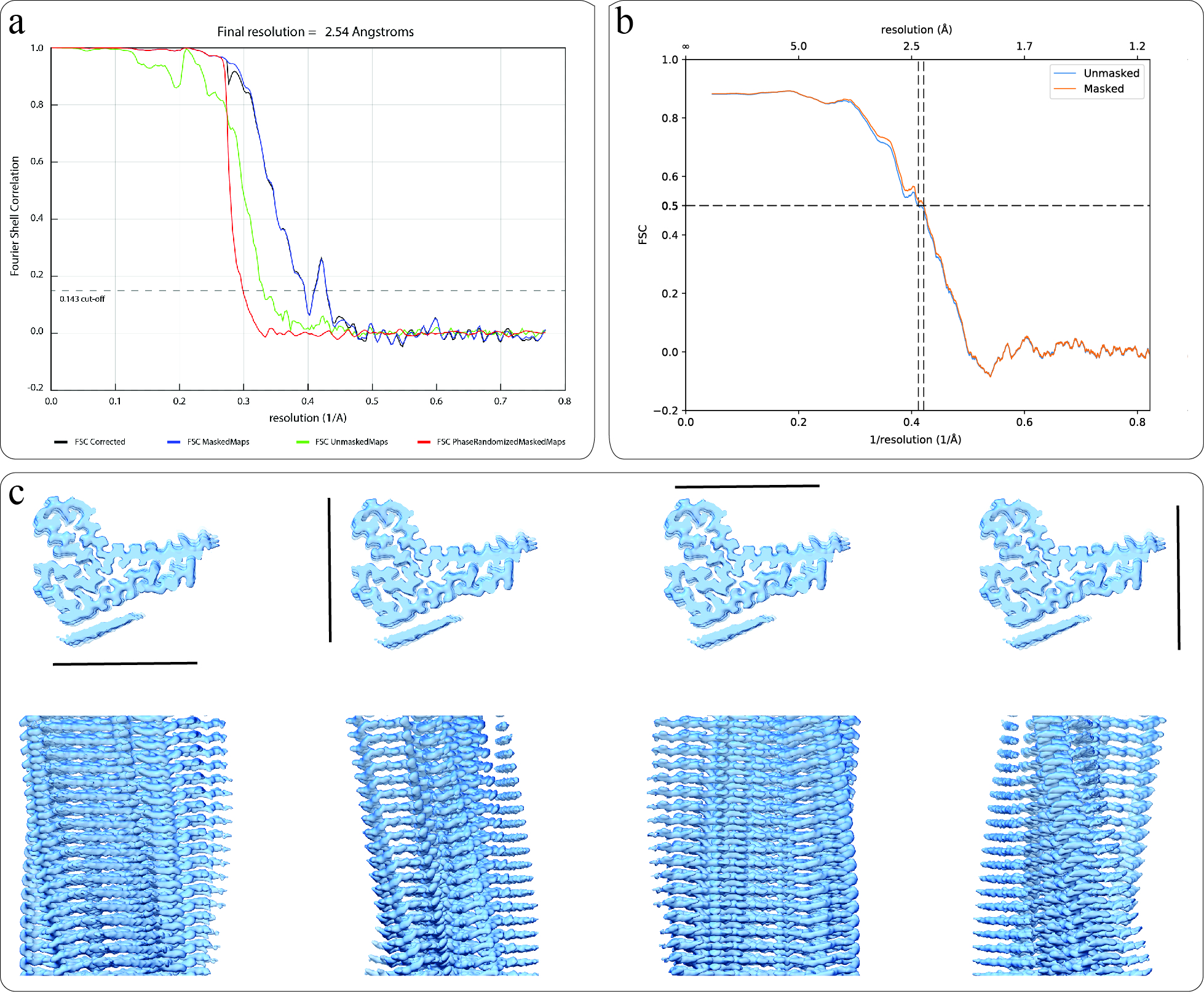


Fig. S1. Cryo-EM analysis of the 17 kDa alpha-amylase/trypsin inhibitor Type 2 (UniProt ID: AI172). (a) Half-map FSC curves for the final model. (b) Model-map FSC curves for the final 5-layer model against a map cropped to within 3Å of the model. (c) Four side views of the final map showing the resolution in the direction of the helical axis. The black line near the top of the fibril indicates the orientation of the side views below it.

|  | AI172 |
| --- | --- |
| **Data Collection** |  |
| Pixel size [Å] | 0.65 |
| Defocus Range [µm] | -0.8 to -2.5 |
| Voltage [kV] | 300 |
| Number of frames | 40 |
| Total dose [e- /Å^2^] | 62.79 |
| **Reconstruction** |  |
| Reconstruction Box width [pixels] | 512 |
| Inter-box distance [Å] | 33 |
| Reconstruction Pixel size [Å] | 0.65 |
| Micrographs | 926 |
| Initially extracted segments | 1,836,771 |
| Segments after 2D classification | 140,376 |
| Segments after 3D classification | 121,923 |
| 3D refinement Resolution [Å] (FSC > 0.143) | 3.02 |
| Final resolution [Å] (FSC > 0.143) | 2.54 |
| Estimated map sharpening B-factor [Å^2^] | -49.24 |
| Axial symmetry | C1 |
| Helical rise [Å] | 4.73 |
| Helical twist [°] | -2.28 |
| **Model composition and validation** |  |
| Non-hydrogen atoms (5 layers) | 2360 |
| Protein residues (5 layers) | 330 |
| R.m.s. devations bond length [Å] | 0.004 |
| R.m.s. devations bond angles [°] | 0.988 |
| MolProbity score | 1.34 |
| Clashcore | 3.25 |
| Rotamer outliers [%] | 0 |
| Ramachandran plot favored [%] | 96.56 |
| Ramachandran plot allowed [%] | 3.44 |
| Ramachandran plot disallowed [%] | 0 |
| Model Resolution [Å]  FSC threshold | 2.1 / 2.4  0.143 / 0.5 |
| PDB code | 9QLU |
| EMDB-ID | 53234 |
|  |  |

**Table S1: Cryo-EM structure determination and model statistics**
